# The value of social interactions and incentives on the use of a digital contact tracing tool post COVID-19 lockdown in Singapore

**DOI:** 10.1038/s41598-022-16820-0

**Published:** 2022-07-20

**Authors:** Zhilian Huang, Huiling Guo, Hannah Yee-Fen Lim, Kia Nam Ho, Evonne Tay, Angela Chow

**Affiliations:** 1 Department Department of Preventive and Population Medicine, Tan Tock Seng Hospital, 11 Jalan Tan Tock Seng, Singapore, 308433 Singapore; 2 Nanyang Business School, Nanyang Technological University, Singapore, Singapore; 3 Lee Kong Chian School of Medicine, Nanyang Technological University, Singapore, Singapore

**Keywords:** Infectious diseases, Health care economics, Health policy

## Abstract

We assessed the preferences and trade-offs for social interactions, incentives, and being traced by a digital contact tracing (DCT) tool post lockdown in Singapore by a discrete choice experiment (DCE) among 3839 visitors of a large public hospital in Singapore between July 2020 – February 2021. Respondents were sampled proportionately by gender and four age categories (21 – 80 years). The DCE questionnaire had three attributes (1. Social interactions, 2. Being traced by a DCT tool, 3. Incentives to use a DCT tool) and two levels each. Panel fixed conditional logit model was used to analyse the data. Respondents were more willing to trade being traced by a DCT tool for social interactions than incentives and unwilling to trade social interactions for incentives. The proportion of respondents preferring no incentives and could only be influenced by their family members increases with age. Among proponents of monetary incentives, the preferred median value for a month’s usage of DCT tools amounted to S$10 (USD7.25) and S$50 (USD36.20) for subsidies and lucky draw. In conclusion, DCE can be used to elicit profile-specific preferences to optimize the uptake of DCT tools during a pandemic. Social interactions are highly valued by the population, who are willing to trade them for being traced by a DCT tool during the COVID-19 pandemic. Although a small amount of incentive is sufficient to increase the satisfaction of using a DCT tool, incentives alone may not increase DCT tool uptake.

## Introduction

Unprecedented public health measures have been implemented since the Coronavirus disease 2019 (COVID-19) emerged in 2020. Measures such as border closures, social restrictions (e.g., closure of workplaces, social distancing, mask-wearing in public), and area lockdowns were necessary to control COVID-19 as health systems struggled to cope with a surge in healthcare demand^1,2^. Large-scale containment measures have also lessened the load on contact tracing efforts by rapidly breaking chains of COVID-19 transmissions^2^. Contact tracing is essential in identifying the close contacts of a person with a communicable disease, but conventional methods can be laborious, time-consuming, and subject to recall biases^3^.

Although social restrictions were successful in reducing COVID-19, protracted lockdowns are undesirable as restrictions on human activities can negatively impact the economy and the mental health of populations^4,5^. Digital contact tracing (DCT) apps were initially considered as the panacea to ease social restrictions by improving contact tracing capabilities during the COVID-19 pandemic. However, sustained usage of DCT tools by a critical mass of the population (i.e., 60% – 80%) is required for DCT technologies to successfully complement conventional contact tracing^6^. In reality, population wide DCT implementation was fraught with challenges. Among the myriad of concerns and misconceptions of the technology, concerns on data privacy and data protection, such as what information is collected and who has access to the data were barriers in the adoption of DCT in many countries^7,8^. Singapore’s DCT was well designed in that it was fully data privacy preserving. Other challenges include technology adoption in the elderly population^9^ and the perceived necessity and reliability of DCT tools in contact identification^10,11^.

Misconceptions of DCT tools often arose from a lack of understanding of the “tracing” technology. Bluetooth technology was the preferred architecture for DCT tools during the COVID-19 pandemic^12^. Unlike the Global Positioning System, Bluetooth technologies employ low-energy communication to exchange signals with nearby devices without tracking user location. In the Singapore DCT, anonymous IDs are generated every 15 min from the Bluetooth exchanges and stored locally on the device for a short period. The data is only uploaded to the central database if and when a user is confirmed with COVID-19. The system thus provides maximum protection for individuals’ data.

Studies have employed stated preference methods to predict preferred attributes of DCT apps in specific populations^13–16^. While these predictions provide a good overview of population preferences for DCT apps, the scenarios were largely hypothetical without the population experiencing social restrictions or using a DCT tool. Furthermore, studies have not assessed the willingness of populations to trade being traced by a DCT tool for incentives and social interactions during an ongoing pandemic. A German study found that monetary incentives can increase the uptake of DCT tools^17^, but the trade-off between incentives and social interactions have not been assessed on the use of such tracing technologies.

Singapore developed a national DCT tool – “TraceTogether” (available as an app or a token) and actively implemented it after a two-month partial lockdown in June 2020. “TraceTogether” utilizes Bluetooth technology. Adoption rates increased from 40% in July 2020 to close to 90% in February 2021 after a slew of measures such as token distributions and mandatory check-ins to public venues (such as grocery stores, shopping centres, hospitals, and schools) using the “TraceTogether” app or token^9^. Under the real-life conditions of social restrictions and promoted use of the DCT tool during an ongoing pandemic, we assessed the preferences and trade-offs for social interactions, incentives, and being traced by a DCT tool among different segments of the Singapore population. We also assessed the influence of different types of incentives and significant others on the uptake of the DCT tool.

## Results

The mean age of the respondents was 50 (± 16.8) years, with half having attained tertiary-level education (Table 1). Although 76.6% (2940/3839) reported that they were willing to use/carry the TraceTogether app or token, 57.2% (2194/3839) were using/carrying the DCT at the time of the survey.

**Table 1:** Utility coefficients of attributes from the Discrete Choice Experiment.

	*N* (%), Mean (SD)	Utility coefficient of attributes (95% CI)		
		Social Interactions	Being traced by a DCT tool	Incentives
**Main effects**	3839 (100)	1.45 (1.01, 1.89)**	− 0.04 (− 0.39, 0.30)	0.14 (− 0.19, 0.48)
**Gender**				
Male	1938 (49.9)	0.46 (0.26, 0.66)**	0.16 (0.02, 0.31)*	0.12 (− 0.04, 0.27)
**Age (21—80 Years Old)**				
Mean (SD)	50 (16.8)	− 0.007 (− 0.014, 0.000)*	0.003 (− 0.008, 0.002)	0.003 (− 0.008, 0.002)
**Education level **^**a**^				
Tertiary	2048 (53.4)	0.17 (− 0.06, 0.40)	− 0.03 (− 0.20, 0.14)	− 0.16 (− 0.34, 0.02)
**Willingness to use TraceTogether **^**b**^				
Willing to use	2940 (76.6)	0.11 (− 0.12, 0.35)	− 0.71 (− 0.91, − 0.51)**	0.34 (0.17, 0.51)**
**Use of TraceTogether **^**c**^				
Using TraceTogether	2194 (57.2)	0.18 (− 0.05, 0.40)	− 0.44 (− 0.60, − 0.28)**	0.42 (0.24, 0.60)**
**TraceTogether data security **^**d**^				
Believed that data collected by TraceTogether data is secure	2451 (63.8)	0.46 (0.25, 0.67)**	− 0.40 (− 0.55, − 0.24)**	0.27 (0.11, 0.42)**

Panel conditional fixed logit analysis of a three-attribute discrete choice experiment, adjusted with covariates.

a Tertiary: Diploma and above.

b Willing: Respondents “agreed” or “strongly agreed” that they are willing to use the TraceTogether app or token.

c Using TraceTogether: Respondents indicated that they were using TraceTogether at the point of the survey.

d: Respondents “agreed” or “strongly agreed” that they believed that the data collected by TraceTogether is secure.

*p < 0.05.

**p < 0.001.

### Outcomes of discrete choice experiment

Social interactions (1.45, 95% CI 1.01–1.89) was associated with a higher positive satisfaction score than incentives (0.14, 95%CI − 0.19–0.48), whereas being traced by a DCT tool was associated with a negative satisfaction score (− 0.04, 95%CI − 0.39–0.30).

#### Satisfaction from Social interactions post COVID-19 lockdown

Respondents who were males orr who believed that the data collected by TraceTogether was secure derived significantly higher satisfaction from having social interactions post lockdown, compared with their female counterparts orthose who did not believe that the data collected by TraceTogether was secure, respectively. The satisfaction derived from social interactions also significantly decreased with increasing age.

#### Dissatisfaction from being traced by a DCT tool

Respondents who were females, those who were willing to use/carry the TraceTogether app/token, who were using/carrying the TraceTogether app/token, or those who believed that the data collected by TraceTogether is secure derived significantly lower satisfaction from being traced by a DCT tool.

#### Satisfaction from Incentives provided for the use of a DCT tool

Respondents who were willing to use/carry TraceTogether, who were using/carrying the TraceTogether app/token, or who believed that the data collected by TraceTogether was secure derived significantly higher satisfaction from incentives provided for the uptake of a DCT tool.

**Figure 1:**
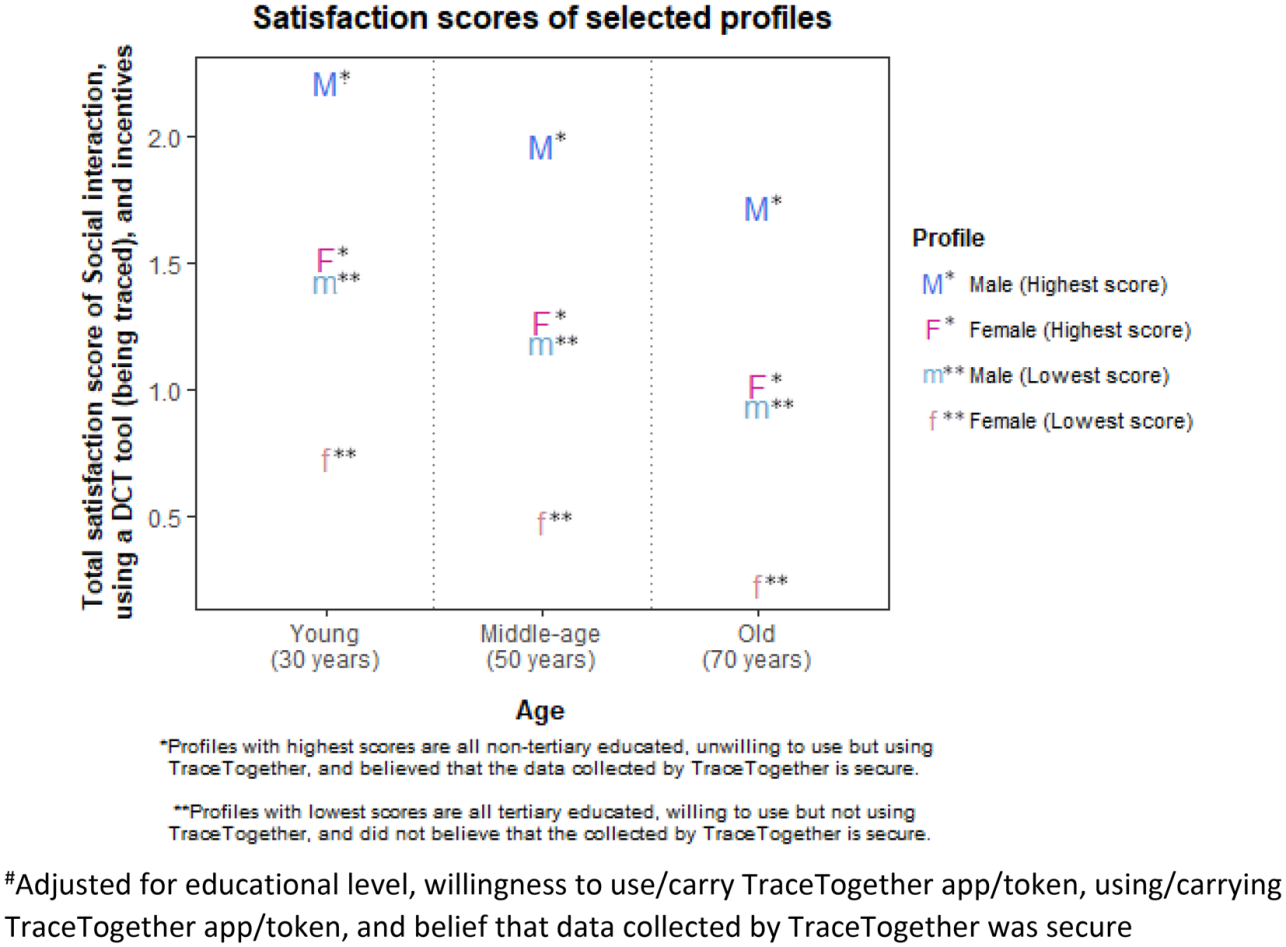
Highest and lowest total satisfaction scores for social interactions and incentives while being traced by a DCT tool, by age and gender^#^.

The total satisfaction scores for social interactions and incentives despite being traced by a DCT tool was computed by summing the utility coefficients of all three attributes for various combinations of the covariates found in Table 1. Ages 30, 50, and 70 were selected for the continuous age variable to represent young, middle-age, and older adult profiles. We computed the satisfaction scores of 96 profiles based on Table 1 and illustrated the differences between the highest and lowest total satisfaction scores for each of the gender and age profiles (Fig. 1).

Young (aged 30 years) males who were non-tertiary educated, unwilling to use/carry the TraceTogether app/token but who were using/carrying the app/token, and who believed that the data collected by TraceTogether was secure derived the highest satisfaction from social interactions and incentives despite being traced by a DCT tool. In contrast, older (aged 70 years) females who were tertiary educated, willing to use/carry the TraceTogether app/token but were not using/carrying the app/token, and who did not believe that the data collected by TraceTogether is secure derived the least satisfaction from social interactions and incentives.

### Trade-offs in satisfaction

**Figure 2:**
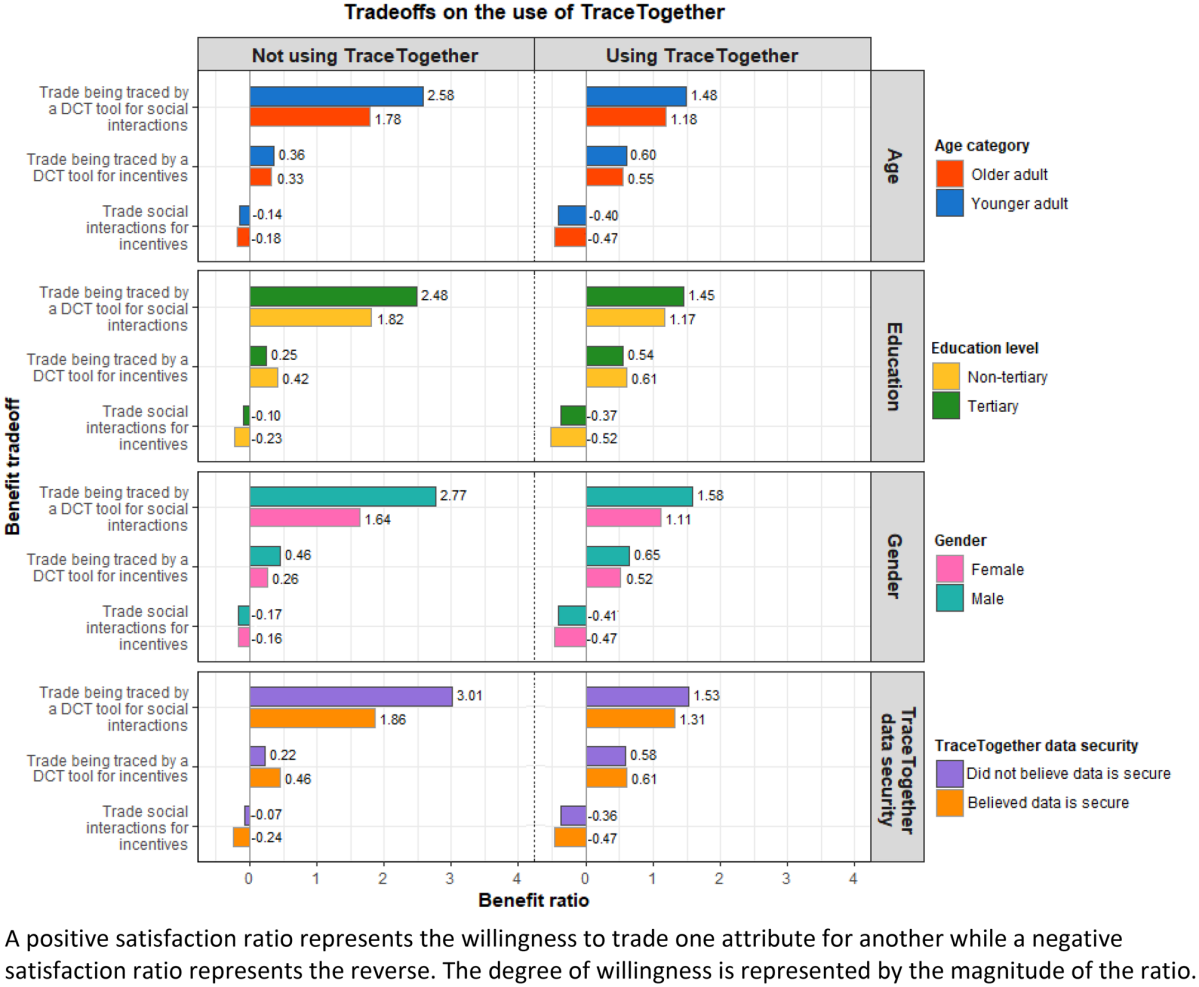
Satisfaction derived from trade-offs between social interactions, incentives, and being traced by a DCT.

A positive satisfaction ratio represents the willingness to trade one attribute for another while a negative satisfaction ratio represents the reverse (Fig. 2). The degree of willingness is represented by the magnitude of the ratio. In general, respondents were more willing to trade being traced by a DCT tool for social interactions than for incentives and were unwilling to trade social interactions for incentives.

Regardless of whether they were using/carrying the TraceTogether app/token, younger adults were more willing than older adults to trade being traced by a DCT tool for social interactions and incentives. Similar preferences were also observed for tertiary-educated respondents compared with non-tertiary educated respondents, and males compared with females. Interestingly, respondents who did not believe that the data collected by TraceTogether was secure were more willing to trade being traced by a DCT tool for social interactions, but more unwilling to trade social interactions and being traced by a DCT tool for incentives than those who believed that the data was secure.

### Incentives and social influence on the uptake of a DCT tool

Table 2 shows the types of incentives and the classes of social influencers that can spur the use of a DCT tool. We classified the classes of social influencers into internal (i.e., Spouse, family members, relatives) and external (i.e., friends, colleagues/classmates, religious leaders) influencers. Persuasions from internal influencers were expected to have longer lasting impact, while those from external influencers more transient effect due to stronger bonds from the familial ties^18^.

**Table 2:** Incentives and social influence on the uptake of a Digital Contact Tracing (DCT) tool.

	^a^ Type of Incentive (*N* = 3915), *n* (%)					^b^ Type of social influence (*N* = 3925), *n* (%)				
	None	Monetary	Lucky draw	Virtual ^c^	*P*-value	Nobody	Internal influence only	External influence only	Internal and external influence	*P*-value
**Age category**										
21–35	193 (19.7)	680 (69.3)	55 (5.6)	53 (5.4)	** < 0.001**	350 (35.7)	140 (14.3)	134 (13.7)	357 (36.4)	** < 0.001**
36–50	255 (26.0)	614 (62.7)	54 (5.5)	56 (5.7)		421 (42.9)	159 (16.2)	131 (13.3)	271 (27.6)	
51–65	403 (41.2)	470 (48.1)	61 (6.2)	44 (4.5)		407 (41.5)	233 (23.8)	139 (14.2)	201 (20.5)	
66–80	549 (56.2)	328 (33.6)	70 (7.2)	30 (3.1)		394 (40.1)	283 (28.8)	162 (16.5)	143 (14.6)	
**Gender**										
Male	678 (34.7)	1033 (52.8)	146 (7.5)	98 (5.0)	**0.03**	791 (40.4)	400 (20.4)	291 (14.9)	476 (24.3)	0.757
Female	711 (36.5)	1059 (54.3)	94 (4.8)	85 (4.4)		781 (39.7)	415 (21.1)	275 (14.0)	496 (25.2)	
**Education level**										
Non-tertiary	852 (46.3)	784 (42.6)	118 (6.4)	85 (4.6)	** < 0.001**	719 (46.7)	477 (31.0)	275 (17.9)	67 (4.4)	** < 0.001**
Tertiary	548 (26.4)	1308 (63.0)	122 (5.9)	98 (4.7)		853 (41.0)	338 (16.2)	291 (14.0)	599 (28.8)	
**Use of TraceTogether**										
Not using TraceTogether	600 (35.4)	923 (54.5)	104 (6.1)	67 (4.0)	0.259	790 (46.6)	369 (21.8)	209 (12.3)	327 (19.3)	** < 0.001**
Using TraceTogether	800 (36.0)	1169 (52.6)	136 (6.1)	116 (5.2)		782 (35.1)	446 (20.0)	357 (16.0)	645 (28.9)	

Significant values are in [bold].

^a^ Respondents were asked to choose the type of incentive that can motivate people to use a DCT tool.

^b^ Respondents were asked to choose from a list of people who could persuade them to use a DCT tool. Internal influence refers to spouse, family members and relatives while external influence refers to friends, colleagues, religious leaders.

^c^ Virtual incentives refer to incentives such as virtual badges, motivational messages on respondents’ “good deeds”, and encouragement on reaching certain milestones on app usage.

Younger respondents aged 21 – 35 years most preferred monetary rewards and two-third (64.4%) could be persuaded by either internal or external social influencers to use a DCT tool. The proportion of respondents preferring no incentives and could only be influenced by their internal social influencers increased with age. Lucky draw and virtual incentives (i.e., virtual badges, motivational messages on respondents’ “good deed”) were least preferred by respondents of all age groups.

A significantly larger proportion of females preferred not to have incentives (36.5% vs. 34.7%) or monetary incentives (54.3% vs. 52.8%) for the use of TraceTogether compared with males. In terms of educational level, a significantly larger proportion of tertiary educated respondents preferred monetary rewards over other incentives (63.0% vs. 43.6%) and could be persuaded to use a DCT tool by both their internal and external social influencers (28.8% vs. 4.4%) compared with non-tertiary educated respondents. There were no significant differences in the preferred type of incentives between current user and non-users of TraceTogether, but a significantly larger proportion (64.9%) of TraceTogether users than non-users (53.4%) could be influenced by their internal and/or external social influencers to use a DCT tool.

Among respondents who preferred monetary incentives, the median value for awards and subsidies amounted to S$10 (USD7.25) while the median value for a lucky draw amounted to S$50 (USD36.20) for the use of a DCT tool for one month.

## Discussion

We assessed the preferences and trade-offs between social interactions, incentives, and being traced by a DCT tool among visitors of a large public hospital on the uptake of the national DCT tool, “TraceTogether”, post COVID-19 lockdown in Singapore. Our study provided invaluable insights into the understanding of population preferences on social interactions and incentives despite being traced by a DCT tool, to increase the uptake of DCT tools during a pandemic amidst the controversies surrounding tracing technologies overseas^19,20^. To our knowledge, this is the first study assessing the trade-offs between social interactions and the use of a DCT tool in a population that has experienced social restrictions due to an ongoing pandemic.

In general, respondents were willing to trade being traced by a DCT tool for social interactions and incentives but unwilling to trade social interactions for incentives. Social interactions in this context refers to external social interactions (i.e., friends, colleagues/classmates, religious leaders), which were highly valued due to the negative impact of lockdowns on the mental health of populations^5,21,22^. Simultaneously, social restrictions and policy mandates (e.g., TraceTogether check-ins at public venues) had accelerated technology adoption^23^, especially among older adults in Singapore^24^. The promoted use of TraceTogether and fatigue with social restrictions may have spurred some individuals to trade social interactions for being traced by a DCT tool. Other studies have also found that populations were willing to use a DCT tool in a pandemic for the benefit of mitigating the pandemic^7^.

Males, younger, and tertiary educated adults placed a higher value on social interactions and were more willing to trade being traced by a DCT tool for social interactions, compared with females, non-tertiary educated, and older adults. This finding was not surprising as men tend to form wider social networks than women^25,26^. Older adults may have a lower preference for social interactions during the pandemic due to higher risks of severe COVID-19 disease or for the public good in response to the government’s call to minimize social interactions in the containment of the pandemic. Studies have shown that older adults display more altruistic behaviors compared with their younger counterparts in caring about the welfare of others^27^. This observation is corroborated with another significant finding by our study that the perception that no incentive was required to motivate the public to use a DCT tool increased with age. Regarding the preferred type of incentive and social influence, there appears to be a correlation between education level and age categories and this can be explained by the fact that older adults in Singapore were less well educated than younger adults^28^.

Respondents who did not believe that the data collected by TraceTogether was secure were more willing to trade being traced by a DCT tool for social interactions compared with those who believed that the data was secure. Among respondents who did not believe that the data was secure, non-users of TraceTogether were more willing than users to trade being traced by a DCT tool for social interactions, suggesting that factors beyond concerns about being traced may have discouraged the uptake of TraceTogether. Since respondents valued social interactions highly, the prospect of social restrictions relaxation may influence the uptake of TraceTogether among non-users.

Our DCE analysis demonstrated unique preferences across respondent profiles, which implied the need for targeted interventions to improve the uptake of DCT tools. For example, younger adults may be interested in referring the DCT tool to people within their social circles and may be further incentivized with a small amount of monetary rewards, while education on technology usage and encouragement from family members may improve the uptake of a DCT tool among older adults^29^. Since TraceTogether users derive higher satisfaction from incentives than non-users, a small amount of incentive (USD 7.25) could help to sustain the usage of such tools.

Limitations exist in this study. We could not quantify the amount of monetary incentives required to trade being traced by a DCT tool. The inclusion of more attributes and levels could have allowed us to do so, but we had kept the choice sets simple to minimize respondents’ fatigue and to encourage a higher participation rate. Our results were also limited by the evolving COVID-19 situation in Singapore. During data collection period, the population uptake of TraceTogether more than doubled due to a slew of promotional messages and enforcements to increase the uptake of TraceTogether. Hence, respondents’ preferences might not be consistent over time. Nevertheless, the overall direction of satisfaction trade-offs between the three attributes should still be consistent during the post lockdown period.

In conclusion, social interactions are highly valued by the population, who are willing to trade them for being traced by a DCT during the COVID-19 pandemic. Although a small amount of incentive (USD 7.25) is sufficient to increase the satisfaction of using a DCT tool, incentives alone may not increase the uptake of DCT tools. Discrete choice experiments can be used to elicit profile-specific preferences to target interventions that can optimize the uptake of DCT tools during a pandemic.

## Methods

### Study design and setting

We conducted a cross-sectional study over a period of eight months post COVID-19 lockdown in Singapore, from July 6, 2020, through February 26, 2021. Up to 160 respondents (patients or their caregivers) were purposively sampled weekly to complete an interviewer-administered questionnaire during their visit to the two busiest ambulatory clinics at the second largest public hospital in Singapore. The respondents were proportionately stratified by gender and the following age categories (in years): 21–35; 36–50; 51–65; 65–80. We included only citizens and permanent residents of Singapore between ages 21 – 80 as this population was the most probable group of people who would fit the context of our study.

### Discrete choice experiments

We conducted a discrete choice experiment (DCE) to elicit respondents’ preferences in the uptake of DCT^30^. The DCE approach is anchored on the utility theory which postulates that when presented with alternatives, a rational individual (who is somewhat self-centred and who does not subscribe to other philosophical thoughts such as virtue ethics) would select the most preferred alternative that maximizes his/her utility (satisfaction or benefit)^31^.

The utility function is defined as:

$${U}_{i}=V\left(\beta , {X}_{i}\right)+{\varepsilon }_{i}$$

McFadden (1973) proposed modelling the expected utilities in terms of characteristics of the alternatives rather than attributes of the individuals^32^. In the equation above, *U*_*i*_ represents the total utility derived from the *i*^*th*^ alternative, β and X_i_ are a vector of estimated coefficients and attribute levels defining the alternative *i*. Each estimated coefficient is a preference weight and represents the relative contribution of the attribute level to the utility that respondents assign to an alternative. The probability of choosing the alternative *i* is equivalent to one alternative *i* among the choice of *j*^*th*^ alternatives^33^.

$$\mathrm{Pr}\left(Choice=i\right)=\frac{{e}^{V(\beta , {X}_{i}) }}{{\sum }_{j}{e}^{V(\beta ,{X}_{i})}}$$

The logit function of a three-attribute study can be simplified as a linear function.

$$\mathrm{Pr}\left(Choice\right)= {\beta }_{0}+{\beta }_{1}{x}_{1}+{\beta }_{2}{x}_{2}+ {\beta }_{3}{x}_{3}$$

Marginal effects can be obtained from the partial derivatives of the attributes. The ratio of coefficients (-β_1_/β_2_) represents the trade-off between two attributes (trading *x*_1_ for *x*_2_) when x_3_ is set to zero.

$$\begin{aligned}\frac{\partial }{\partial x}&=\frac{\partial }{\partial {x}_{1}}\left({\beta }_{0}\right)+\frac{\partial }{\partial {x}_{1}}\left({\beta }_{1}{x}_{1}\right)+\frac{\partial }{\partial {x}_{1}}\left({\beta }_{2}{x}_{2}\right)+\frac{\partial }{\partial {x}_{1}}\left({\beta }_{3}{x}_{3}\right)=0\\ \frac{\partial {x}_{2}}{\partial {x}_{1}}&=-\frac{{\beta }_{1}}{{\beta }_{2}}\end{aligned}$$

### Questionnaire design

We designed a DCE questionnaire with three attributes (1. Social interactions, 2. Traced by a DCT tool, 3. Incentives to use a DCT tool) and two levels (presence or absence of the attribute) in the context of the COVID-19 situation in Singapore in June 2020. The country had just exited a 2-month partial lockdown and the use of “TraceTogether” was widely promoted during the months post lockdown. All eight combinations of the attribute levels were considered and combinations that mirrored each other were paired as a choice set (Table 3).

**Table 3:** Discrete choice experiment choice sets.

Q set	Choice	Attributes			
		Social interaction ^a^	Traced by a DCT tool ^b^	Incentive ^c^	
1	A	Yes	Yes	Yes	Respondents who chose option B do not place a high value on **incentives** and **social interactions and may have concerns on being traced by a DCT tool**
	B	No	No	No	
2	A	Yes	No	No	Respondents who chose option B place a high value on **incentives**
	B	No	Yes	Yes	
3	A	No	No	Yes	Respondents who chose option B place a high value on **social interactions**
	B	Yes	Yes	No	
4	A	Yes	No	Yes	This choice set is a test of rationality. Respondents who chose option B were asked for the reason(s) for their choice
	B	No	Yes	No	

^a^ Social interaction: Ability of the respondent to engage in social activities when a lockdown was not in force.

^b^ Traced by a DCT tool: Whether close contact within 2 m had occurred between 2 devices were captured due to the carrying of a DCT tool. Negative attribute.

^c^ Incentive: Any incentive (e.g., monetary, virtual rewards, lucky draw) which the respondent thought was reasonable to spur him/her to carry a DCT tool and/or to reduce his/her social activities.

DCE indicates discrete choice experiment.

In this context, “Social interactions” refers to the ability of the respondent to engage in social activities when a lockdown was not in force; “Traced by a DCT tool” refers to the capturing of signals of 2 “TraceTogether” devices (app or token) within 2 m of each other due to the respondent’s carrying of a DCT tool; and “Incentives” refers to any incentive (e.g., monetary, virtual rewards, lucky draw) which the respondent thought was reasonable to spur him/her to carry a DCT tool and/or to reduce his/her social activities. The questionnaire was piloted over two weeks with 154 respondents to refine the expression based on feedback from respondents. The questionnaire was also translated into Mandarin Chinese to cater to respondents who could not comprehend the English language.

### Data collection

All data collectors were trained to administer the questionnaire in a standardized manner guided by infographics, to minimize misinterpretation of the survey questions. Respondents were first asked if they were using the “TraceTogether” app or token, their willingness to use the “TraceTogether” tool, and whether they believed the data collected by “TraceTogether” was secured. They were then presented with two hypothetical scenarios for each DCE choice set and asked to choose their preferred option. After the DCE choice sets, respondents were asked the type of incentives that they thought would most likely motivate the population to use a DCT tool and who could persuade themselves to use a DCT tool during a pandemic. If the respondent chooses monetary incentive as their most likely motivation for the population to use a DCT tool, they were asked if a certain amount ($5 incremental) is sufficient to sustain the use of the DCT tool. The respondent was asked to suggest an appropriate amount of money if $50 is insufficient.

Demographic information was also collected to perform segmented analyses. We screened 5973 potential respondents, of which, 689 (11.5%) were not eligible for the study, 1341 (22.5%) refused to participate, and 3943 (66.0%) were interviewed. The dataset comprises 3892 respondents after dropping 51 (1.3%) respondents who failed to complete all the DCE choice sets.

### Analysis

We further removed 53 respondents who did not provide a valid reason for choosing the “irrational” choice (Table 3, Q4, Choice B) and dropped Q4 from the analysis. The remaining 3839 responses (Q1 – Q3) were analysed using the panel fixed conditional logit model with the robust variance estimator to correct for heterogeneity of variance. Akaike’s information criterion (AIC) and Bayesian information criterion (BIC) were computed for the selection of the best model, with a preference for lower AIC and BIC values (Table S2). The variables included in the final model are gender, age, tertiary education, willingness to use “TraceTogether”, using “TraceTogether”, and whether the respondent thought the data collected by “TraceTogether” will be secured.

Segmented analyses were performed to assess attribute trade-offs (by dividing the coefficients of the final model) between sociodemographic groups. We computed the total satisfaction scores of various profiles by adding up the coefficients of individual characteristics to illustrate the preferences between profile groups.

Descriptive analyses were conducted to assess the type of incentives participants thought could most likely motivate the population to use a DCT tool. Lucky draw or intangible items (e.g., points to claim vouchers) were converted to a monetary value based on the average cost of the item in the year 2020 to assess the monetary value of the incentives (Table S3). All analyses were performed with STATA version 15.0^34^ and RStudio version 1.2.5033^35^.

### Sample size

We used the method proposed by de Bekker-Grob et al. to compute the minimum sample size required for this DCE analysis^36^. Initial estimates based on the pilot dataset suggested a sample size of 1481 to detect differences in the main effects at a 0.05 statistical significance and 80% statistical power. Our post hoc analysis revealed that our 3839 responses were sufficient to detect differences in the main effects at 0.01 significance level with a power of 90% (Table S1).

### Ethics approval

Ethics approval was obtained from the National Healthcare Group Institutional Review Board (DSRB: 2020/00,775), in accordance with the relevant guidelines from the Declaration of Helsinki and the ethical principles in the Belmont Report. All participants gave written informed consent.

### Consent for publication

All authors reviewed and approved the final version of the manuscript prior to submission.

## Supplementary Information

Supplementary Information.

## Data Availability

The datasets used and/or analysed during the current study available from the corresponding author on reasonable request.
