# Supplementary material for: The value of social interactions and incentives on the use of a digital contact tracing tool post COVID-19 lockdown in Singapore
Source: Sci Rep. 2022 Jul 20;12:12416. doi: 10.1038/s41598-022-16820-0 (PMC9297674; doi:10.1038/s41598-022-16820-0)
Supplement: Supplementary file 1 — Supplementary Information. [file 41598_2022_16820_MOESM1_ESM.pdf]

Supplementary files

Table S1: Minimum sample size required for Discrete Choice Experiments

Conditions		Initial estimates			Final estimates		
Alpha	1-Beta	Social interaction	Privacy loss	Incentives	Social interaction	Privacy loss	Incentives
0.05	0.8	33	188	**1481**	56	167	**742**
0.05	0.9	46	260	2051	78	231	1027
0.001	0.8	83	470	3702	141	417	1855
0.001	0.9	102	581	4577	175	516	2293

Parameters for initial estimates: (SI, P, I) = (2.303, -1.076, 0.373)
Parameters for final estimates: (SI, P, I) = (1.764, -1.142, 0.527)

Table S2: Steps for model selection

Model	Variables include in the model	AIC	BIC
M1	Main effects (Social interactions, Privacy Loss, Incentives)	15665.2	15689.4
M2	M1 + Gender (Male, Female)	15620.2	15668.4
M3	M2 + Age (21 - 80 Years Old)	15594.3	15666.7
M4	M3 + Education level (Tertiary, Non-tertiary)	15584.9	15681.4
M5	M4 + Employment status (Employed, Unemployed)	15587.1	15707.7
M6	M5 + Willingness to use TraceTogether (Willing, Unwilling)	15215.3	15360.1
M7	M6 + TraceTogether usage (Using, Not using)	15118.6	15287.6
M8	M7 + Believes that TraceTogether data is secure (Secure, Not secure)	15028.6	15221.7
**M9**	M8 - Employment status	**15026.3**	**15195.3**

M9 was chosen as the final model as it has the lowest AIC (Akaike Information Criteria) and BIC (Bayesian Information Criteria), which indicates better model fit.

Table S3: Monetary value of incentives quoted by respondents for the uptake of a DCT tool

Lucky Draw Items		
**Desired rewards mentioned by respondents**	**Estimated cost based on 2020 consumer Price Index, S$ (USD)**	**Justification**
Apple phone/Handphone/Phone	1,200 (869.60)	Based on prices of Apple and Android phone models sold by local retailers
Cab rides	9 (6.50)	Based on standard taxi flag down fares in Singapore during non-peak periods
Car	100,000 (72,463.80)	Average cost of a car in Singapore
Central Provident Fund top-up	500 (362.30)	Monthly sum given to citizens affected by COVID-19
COVID-19 vaccination/ (Customized) TraceTogether token	0 (0)	Free for all Singaporeans and Permanent Residents
Daily essentials/necessities	25 (18.10)	Cost of daily essentials care package given out in national campaigns
Digital/Electronic devices/	400 (289.90)	Estimated based on similar devices commonly given out as lucky draw prizes
Electrical appliances/Household items	500 (362.30)	Based off value of similar devices commonly given out as lucky draw prizes
Grocery store membership cards	10 (7.20)	Estimated from membership costs for NTUC and similar grocery stores
Health/Medical check-ups	200 (144.90)	Cost of basic health screening package at public hospitals in Singapore
Healthcare products (e.g., health supplements/medical goods)	50 (36.20)	Approximated from prices of health products sold at public hospital pharmacies.
Lottery prize/Scratch cards	50 (36.20)	Median value of lucky draw specified by respondents preferring "lucky draw"
Miscellaneous items (e.g., Containers/Food/Keychains/Soft toys)	5 (3.60) - 10 (7.20)	Estimated from cost of such products in retail stores
Plane tickets	900 (652.20)	Mean cost of tickets for return ticket to the most popular destination
Portable charger/Power bank	60 (43.50)	Estimated Price in Singapore
SingapoRediscover vouchers	100 (72.50)	Local travel vouchers awarded by the government to each Singapore citizen
Staycation	300 (217.40)	Average cost of 5-star hotel stays for one night in Singapore
Surgical masks/Masks	15 (10.90)	Approximated from prices of 50pc boxes of surgical masks in Singapore
Television	1,600 (1,159.40)	Based on prices of popular television models
Unspecified items (e.g., “Dependent on demographic”/ “whichever”)	50 (36.20)	Median value of lucky draw specified by respondents preferring "lucky draw"
Wi-Fi nationwide	0 (0)	Wi-Fi is free nationwide at hotspots

Monetary Subsidies		
**Desired rewards mentioned by respondents**	**Estimated cost based on 2020 consumer Price Index, S$ (USD)**	**Justification**
20% off government tax or bills	300 (217.40)	20% of gross payable tax paid by residents of Singapore according to median gross monthly income
Credit for government programmes (e.g. ActiveSG/Skillsfuture)	100 (72.50)	Value of skill upgrade credits given to Singapore adult citizens
Central Provident Fund top-up (Specified value)	200 (144.90)	In accordance with value specified by respondents
Data/Mobile subcription rebates	5 (3.60)	Based off the Mobile Access for Seniors scheme
Deduct fees for service and conservancy charges	140 (101.40)	Based on amount of service and conservancy charges rebate each household received
Healthcare benefits like free treatment if tested positive for COVID-19	0 (0)	Treatment for COVID-19 is free for all Singaporeans and Permanent Residents
Medical cash value	200 (144.90)	Approximated from amounts offered by Singapore insurance companies
Medical discount vouchers/benefits/subsidies	63 (45.7)	Mean subsidy per consultation at public hospitals in Singapore
Mobile data	20 (14.50)	Estimated from prices of basic data-only plans
Money for Medisave	78 (56.50)	Median sum contributed monthly to Medisave by each citizen based on minimum eligible salary
PAssion membership card top-up	20 (14.5) - 50 (36.2)	In accordance with values specified by respondents
Point system (e.g., Accumulate points/Air miles/Health Promotion Board points)	5 (3.60)	Based off existing initiative by Singapore's Health Promotion Board that utilizes a point system
Tax rebates	1,000 (724.60)	Amount of earned income relief offered to individuals employed in Singapore because of COVID-19
Unspecified amount (e.g., Money/Public transport subsidy/Transport rebates)	5 (3.60)	Minimum sum researchers posed to respondents who chose monetary rewards
Utilities bill rebates	240 (173.90)	Mean amount of rebate granted to each household
Utilities bill vouchers 20%	48 (34.80)	In accordance with value specified by respondents
Vouchers (e.g., Gift vouchers/Grocery vouchers)	2 (1.40) - 500 (362.30)	In accordance with values specified by respondents
Work compensation for people on home quarantine order	0 (0)	Provided to all employees under work injury compensation and employment act

*Items that were given free of cost to all citizens are coded as $0 as participants do not have to put in extra effort to earn the incentive.
